# Effectiveness of a Family Support Intervention on Caregiving Burden in Family of Elderly Patients With Cognitive Decline After the COVID-19 Lockdown

**DOI:** 10.3389/fpsyt.2021.590104

**Published:** 2021-03-04

**Authors:** Luca Cravello, Eleonora Martini, Niccolò Viti, Cristina Campanello, Francesca Assogna, Daniele Perotta

**Affiliations:** ^1^Alzheimer Center ASST Rhodense, Rho, Italy; ^2^Fondazione Santa Lucia, Istituto di Ricovero e Cura a Carattere Scientifico (IRCCS), Rome, Italy

**Keywords:** dementia, Alzheimer's disease, caregiver, behavioral disorders, non-pharmacological treatment, neuropsychiatric symptoms, psychoeducational interventions, burden

## Abstract

**Background:** The coronavirus disease 2019 (COVID-19) pandemic had a great impact on patients with cognitive decline or dementia. The lockdown period may exacerbate behavioral disorders and worsen distress of caregivers. The aim of this study is to evaluate the effectiveness of a family support intervention on the negative effects that the COVID-19 lockdown may have on patients and related caregivers.

**Methods:** We recruited patients whose related caregivers had attended a family support course before the COVID-19 lockdown. The course was for family members of patients with cognitive decline or dementia and consisted in eight meetings during which the participants received information about the disease, the management of neuropsychiatric symptoms, and community resources and services available for patients with dementia. Data on cognitive decline, neuropsychiatric symptoms, and functional status had been collected before the course with the Mini-Mental State Examination (MMSE), the Neuropsychiatric Inventory (NPI), and the Instrumental (IADL) and Basic (BADL) Activities of Daily Living scales, respectively. The caregiving burden had been evaluated at the end of the course by means of the Zarit Burden Interview (ZBI). After the COVID-19 lockdown, a phone interview was made to compare neuropsychiatric symptoms, functional status, and caregiver's burden with the previous evaluation.

**Results:** There were no significant changes before and after the COVID-19 lockdown in the mean NPI score. The IADL, BADL, and ZBI scores were significantly lower after lockdown than before. The BADL scores were inversely associated with ZBI scores. Thus, despite a worsening of patients' functional status, the caregivers' burden decreased significantly probably due to the positive effect of the family support intervention.

**Conclusions:** Our study demonstrated that a complete family support intervention for caregivers of patients with cognitive decline or dementia can reduce the burden of care even in a particular negative period, such as the COVID-19 lockdown.

## Introduction

The coronavirus disease 2019 (COVID-19) pandemic had a great impact on the global population. Among them, older people are still paying the higher price in terms of mortality, probably because of underlying chronic illnesses, such as hypertension, diabetes, ischemic heart disease, chronic obstructive pulmonary disease, and hematological and oncological diseases, which represent the main dangerous factors of this infection (1). The high risk of mortality following COVID-19 infection in the elderly population is now well-known, whereas the impact that the lockdown restrictive measures have had on the general population and on frail elderly people in particular is not yet completely clear.

In fact, during the lockdown period, the whole Italian population was confined at home, the normal daily routine was completely disrupted, no longer allowing regular motor activity and social interactions that for many elderly people were of fundamental importance. In addition, many facilities for the elderly were no longer able to provide their services: the day centers in the residential facilities were closed, and community resources were temporarily interrupted.

It was observed that the COVID-19 lockdown-related restrictions had a great impact on patients with mild cognitive impairment (MCI) or dementia (2) with worsening of the behavioral and psychological symptoms of dementia, also called neuropsychiatric symptoms (3, 4). Neuropsychiatric symptoms are a group of heterogeneous symptoms that include psychosis, agitation/aggression, dysphoria, anxiety, euphoria, apathy, disinhibition, irritability/lability, aberrant motor activity, night-time behavioral disturbances, and appetite and eating abnormalities (5). Neuropsychiatric symptoms occur at all stages of cognitive disorders including pre-clinical, MCI, or dementia (6) and are associated with more rapid cognitive decline and poor functional status (7). Neuropsychiatric symptoms are a frequent reason for institutionalization (8), have a high prevalence in residents of long-term care homes (9), are associated with increased mortality risk, and cause considerable suffering for individuals with dementia and their caregivers (10). Moreover, neuropsychiatric symptoms are the most stressful aspects strongly reducing the quality of life for both patients and caregivers (11), leading to medical interventions, changes in pharmacological therapy, and greater use of drugs potentially harmful for the elderly, such as neuroleptics and benzodiazepines (12, 13).

Two recent studies found worsening of neuropsychiatric symptoms in patients with MCI or Alzheimer's disease (AD) during the COVID-19 confinement (3, 4). Moreover, the duration of confinement was significantly correlated with the severity of symptoms as well as with the caregivers' distress (3). Another sub-study of a multicenter nation-wide survey based on a structured telephone interview delivered to family caregivers of patients with AD or other types of dementia found an increased prevalence of symptoms of anxiety, feeling of helplessness, anguish, concern for patient health, and familial conflicts reported by caregivers after COVID-19 quarantine (14).

Caregivers of persons with dementia commonly show high levels of psychological distress and burden related to the emotional involvement and to the comprehension and acceptance of a disease that currently does not have a specific cure (15); moreover, most of the caregivers are not prepared to deal with neuropsychiatric symptoms requiring guidance on where and how to get practical help and some advice. Several data in the literature support the efficacy of specific interventions in reducing neuropsychiatric symptoms in related caregiver's burden. A recent systematic review and meta-analysis found that psychoeducational programs focused on improving management and problem-solving in difficult situations lead to a significant reduction of the level of stress and burden of the caregivers (16). Moreover, some data suggest that individual psychoeducational interventions have the strongest effects (17), whereas more recent findings suggest better efficacy of group interventions (15).

It has been highlighted that the COVID-19 quarantine can determine neuropsychiatric symptoms increase in patients with dementia and higher burden of their caregivers (14, 18). A recent study found that 51.9% of patients with dementia had worsening of preexisting neuropsychiatric symptoms, 26% of patients had new neuropsychiatric symptoms onset, and 27.6% of patients requested drug modifications related to neuropsychiatric symptoms. Moreover, stress-related symptoms and increased burden were experienced by two-thirds of caregivers (14) during the quarantine.

Considering that specific psychoeducational interventions can reduce the stress of caregivers, the purpose of this study is to evaluate the effectiveness of a family support intervention on the caregivers' burden after the COVID-19 lockdown. In particular, we suppose that the family caregivers who received training on non-pharmacological dementia management strategies are able to address the neuropsychiatric symptoms during the COVID-19 lockdown and thus they have a low level of caregiving burden.

## Methods

### Participants

We collected data from the Alzheimer's Regional Center of the ASST Rhodense, an outpatient memory clinic near Milan (Italy). We included only those patients whose related caregivers participated to the last edition of the course for family members of patients with AD that took place from September to December 2019. Inclusion criteria were: (1) patients with at least one cognitive evaluation within 3 months before the course, (2) caregivers who participated to the first two meetings and at least to the 50% of the course, (3) caregivers able to undergo testing procedures, and (4) caregivers must have, in the judgement of the clinician, frequent and sufficient contact with the patient to be able to provide accurate information regarding the patient's cognitive, behavioral, and functional status: a contact was considered “sufficient” when the caregiver was living with the patient or, in case the patient was living alone (i.e., for patients with MCI), when the caregiver had daily contact with the patient.

Of the 94 patients, 41 were excluded because the caregiver did not participate to at least four of eight meetings. Eight patients were excluded because the caregivers refused to fill out the burden caregiver questionnaire. We also excluded 4 subjects because the caregivers did not attend the first two meetings that were essential to learn important information about the disease and how to handle neuropsychiatric symptoms and problems related to dementia. Finally, we excluded 7 patients because the caregivers did not have sufficient contact with the patients. Thus, the final study population consisted of 34 patients and related caregivers. Each patient and caregiver had signed an informed consent for the use and processing of personal data before participating to the course for family members.

### Family Support Intervention

We propose a family support intervention every year, for the family members of our patients to support them in acquiring and maintaining the difficult caregiver's role. Our family support intervention consists in a training course divided into eight meetings during which the participants receive information on different aspects of the disease, they are instructed on the management of behavioral and psychological symptoms associated with dementia, and they acquire knowledge about public care services and associations for family members in our area.

Each meeting is divided into a part of frontal lesson of about 80 minutes and a part of about 40 minutes in which there is a lot of time for questions and for sharing experiences. At each meeting, one or more speakers (specialists from the memory clinic, psychologists, lawyers, nurses, physiotherapists, representatives of the voluntary association, and general practitioners) take part. Moreover, a specialist from the memory clinic (clinician or psychologist) moderates the discussion and sharing part. The first two meetings are dedicated to the description of the cognitive disease, the different types of dementia, and the related behavioral disorders: the participants are informed on the definition and natural course of the disease, and they are advised on pharmacological therapies and progress in research, cognitive and motor stimulation, prevention, and lifestyles. The third and fourth meetings are focused on illustration, sharing, and training on non-pharmacological interventions of cognitive and behavioral disorders especially at the level of home care management by the caregivers. The fifth meeting is about legal issues related to AD or other forms of dementia, with particular attention to the role of the legal administrator. The topic of the sixth meeting is the severe phase of dementia and the end-of-life care for people with dementia: the principles of intervention and the role of the general practitioner in collaboration with the specialist are debated. In the seventh lesson, a psychologist discusses the theme of the family and AD, giving strategies for best living with people with dementia. Finally, in the last meeting, the local resources for people with dementia are described: Alzheimer's volunteers' groups, Alzheimer's association programs, social resources, and nursing homes. The main family course program is available as Supplementary Material.

At the end of each meeting, data were collected by means of questionnaires administered to family members in order to monitor risk situations and to evaluate the effectiveness of proposed intervention.

### Patients and Family Members Evaluation

For each patient, the following categories of data were collected: demographic data, diagnosis, and level of cognitive decline. All patients must have a complete clinical and neuropsychological evaluation to confirm the diagnosis of cognitive decline and/or dementia. The National Institutes of Health and the Alzheimer's Association (NIA-AA) criteria were used for the diagnosis of MCI (19) and AD (20). Specific international criteria were adopted for other types of dementia (21).

For what concerns the level of cognitive decline, an evaluation in the outpatient memory clinic was performed no more than 3 months before the course for family members. The level of cognitive decline was assessed with the Mini-Mental State Examination (MMSE) (22), the neuropsychiatric symptoms were assessed with the Neuropsychiatric Inventory (NPI) (5), and the level of functioning was assessed with the Instrumental Activity of Daily Living (IADL) scale (23) and the Basic Activity of Daily Living (BADL) scale (24). At the end of the last meeting of the course for family members, the Zarit Burden Interview (ZBI) was administered. The ZBI is an interview for assessing the burden of the caregivers of people with dementia (25). It is a self-report scale that presents self-directed descriptions of management difficulties experienced in the care of a patient with dementia. The ZBI consists of 22 items rated on a 5-point Likert scale that ranges from 0 (never) to 4 (nearly always) with the sum of scores ranging between 0 and 88. Higher scores indicate greater burden.

In May 2020, after 8 weeks of the COVID-19 lockdown, a phone interview with a family member was made to investigate the family's fruition of community resources for dementia before lockdown and health resources during and after lockdown. We interviewed the family member representing the primary caregivers who attended the course (26). In particular, we investigated if patients took part to local community initiatives (i.e., community resources, day care centers, and cognitive training and cognitive rehabilitation groups) before lockdown, if they had COVID-19 infection, hospitalization, and delirium, or if they took advantage of other medical resources (i.e., call to general practitioner, memory clinic, and Alzheimer's associations) during and after the COVID-19 lockdown. During the phone call, the NPI, IADL, BADL and ZBI were also administered.

### Statistical Analysis

The distribution of the analyzed variables was verified using the Shapiro–Wilk test: all continuous variables, except MMSE and age, had a non-normal distribution. Thus, the comparison between pre- and post-lockdown for socio-demographic data and clinical variables was performed using Wilcoxon signed-rank test.

To evaluate the association between caregiver burden during the lockdown and socio-demographic and functional variables, multiple regression analysis was performed. The standardized coefficients (beta) were calculated. ZBI delta score was entered as a dependent variable, whereas delta scores of total NPI, IADL, and BADL (post-lockdown score minus pre-lockdown score), age, sex, and years of education were entered as independent variables. The level of statistical significance was defined as *p* < 0.05.

## Results

In the final sample of 34 patients, 76.5% were female. Mean age ± standard deviation (SD) was 81.5 ± 5.2 years, and mean educational level ± SD was 6.3 ± 2.6 years. Concerning diagnosis, 25 patients (73.5%) had AD, 6 (17.6%) had mixed dementia (AD associated with cerebrovascular disease), 1 (2.9%) had Lewy-body dementia, and 2 (5.9%) were classified as MCI.

The 52.9% of patients were married, and 47.1% were widowed; 70.6% of patients were living with a caregiver, whereas 29.4% of them were living alone. The primary caregivers who attended the course were sons (64.7%), spouses (32.4%), and others (2.9%).

Before the COVID-19 lockdown, 5 patients (14.7%) made use of community resources, 3 patients (8.8%) regularly attended day care center, 1 patient (2.9%) was involved in a cognitive rehabilitation group, and 1 patient (2.9%) benefited from cognitive individual home stimulation.

During the COVID-19 lockdown, only 1 patient (2.9%) had delirium, and 3 patients (8.8%) resorted to clinical care (1 patient referred to a general practitioner, and 2 patients called the outpatient memory clinic). Only for 2 patients, it was necessary to add neuroleptic to the standard pharmacological therapy: no benzodiazepines nor other psychoactive drugs were added. None of the patients got COVID-19 infection nor was hospitalized.

Table 1 shows the pre- and post-COVID-19 lockdown values of clinical variables: our sample of patients had a mean MMSE total score of 16.9 (SD = 5.1, range 3–28). After the COVID-19 lockdown, there was a decrease of NPI total score and NPI caregiver stress score, but it was not statistically significant. Both IADL and BADL total scores were lower after lockdown, indicating a significant functional loss. After lockdown, the ZBI mean score was lower than before, which means a significant lower caregiving burden.

**Table 1:** Clinical variables pre- and post-COVID-19 lockdown.

**Variable name**	**Pre-COVID-19 lockdown**		**Post-COVID-19 lockdown**		***p***
	**Mean**	**SD**	**Mean**	**SD**	
MMSE	16.9	5.1	n.a.	n.a.	
NPI total	13.2	14.9	10.8	15.2	0.136
NPI caregiver stress	6.6	7.1	5.7	7.1	0.284
IADL	3.12	2.3	2.4	2.0	0.005^**^
BADL	5.1	1.3	4.7	1.7	0.013^*^
ZBI	31.6	13.9	25.3	12.9	0.000^***^

*SD, standard deviation; MMSE, Mini-Mental State Examination; n.a., not available; NPI, Neuropsychiatric Inventory; IADL, Instrumental Activity of Daily Living; BADL, Basic Activity of Daily Living; ZBI, Zarit Burden Interview*.

* = p < 0.05;

** = p < 0.01;

*** *= p < 0.001*.

**Figure 1:**
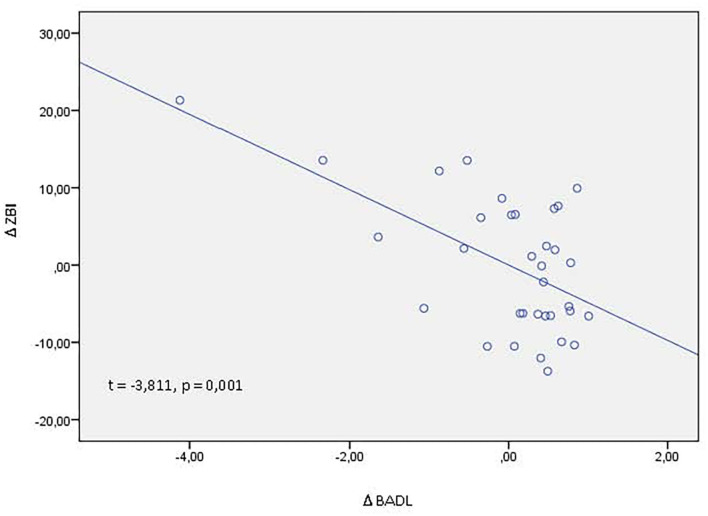
Partial regression plot between Δ ZBI (post-lockdown ZBI score – pre-lockdown ZBI score) and Δ BADL (post-lockdown BADL score – pre-lockdown BADL score). ZBI, zarit burden interview; BADL, basic activities of daily living; *t, t* test statistic; *p*, statistical significance.

In order to evaluate the possible association between ZBI delta score (post-lockdown ZBI score–pre-lockdown ZBI score) and delta score of others clinical variables (post-lockdown score–pre-lockdown score), we performed multiple regression analyses: ZBI delta score was negatively associated with BADL delta score (β = −0.603, *t* = −3.811, *p* = 0.001), meaning that higher disability on basic activities of daily life was associated with higher caregiving burden (Figure 1).

## Discussion

To our knowledge, this is the first study that analyzes the effectiveness of a structured family support intervention on the possible negative effects that the COVID-19 lockdown could have as determined on elderly people with cognitive decline or dementia. We found that, after lockdown, patients whose family members attended the course did not have a worsening of neuropsychiatric symptoms; moreover, the relative caregivers showed less caregiving burden despite a worsening of patients' disability, and finally, there was an extremely low request of medical interventions.

The COVID-19 pandemic had a significant impact on all social and economic sectors worldwide (27); moreover, the general population had to bear the increasing burden of the epidemic with important consequences in terms of psychological impact (28). The elderly with impaired cognition or dementia suffered more the consequences of the COVID-19 lockdown: they could have difficulties in understanding restriction measures, their routine was altered, and social interaction was almost reset; moreover, cognitive stimulation programs were interrupted leading to important alterations in these patients (4), such as the onset or the worsening of neuropsychiatric symptoms. Neuropsychiatric symptoms are described in institutionalized elderly people without dementia (29), in stroke patients (30), in Parkinson's disease (31) and in AD patients, and in other dementia patients (32). They accelerate the progression of disease and institutionalization, and they predict poorer quality of life, increased disability of patients, and great distress for patients and caregivers (33). Lara et al. (4) found worsening of some neuropsychiatric symptoms (agitation, apathy, and aberrant motor activity) after 5 weeks of lockdown in a sample of MCI and AD Spanish population; moreover, many patients and caregivers reported that their health condition had worsened after confinement (4). In another study by Boutoleau-Bretonniere and colleagues, caregivers of 38 patients with AD have been contacted. They were confined to their homes for nearly 2 months, and they were asked to report whether patients experienced any change in neuropsychiatric symptoms during, compared with before, the confinement and rate its severity and impact on themselves using the Neuropsychiatric Inventory—Questionnaire (3). The authors found neuropsychiatric symptom changes during lockdown only in 10 AD patients. For these patients, the neuropsychiatric changes were significantly correlated with symptoms severity and caregivers' distress (3). The lack of neuropsychiatric assessment before the confinement limits the results of the study.

In our study, we did not find a worsening of neuropsychiatric symptoms despite the negative impact that the COVID-19 lockdown could have had on patients with cognitive decline or dementia. We think that this result is to be mentioned and it is likely to be related to the management of neuropsychiatric symptoms learned during the course for family members. Several studies support the efficacy of non-pharmacological treatment, such as sensory stimulation interventions, cognitive/emotion-oriented interventions, behavior management techniques, and other therapies, on neuropsychiatric symptoms in patients with dementia (34). Studies also show that caregiver support, education, training, and skill development finalized to effectively problem solve and communicate can be beneficial (35). In our study, only a quarter of patients attended specific programs that can be considered as non-pharmacological treatment (i.e., day care centers, cognitive rehabilitation groups, and individual cognitive stimulation); thus, we believe that specific information on the disease and the management of behavioral disorders given to family caregivers during the course was powerful in reducing the possible negative effects that the COVID-19 lockdown could have on patients' behaviors. Moreover, the obtained results could be partly attributable also to the full pathway of care proposed in the memory clinic and of which the course belonged to. After the diagnostic process, the patient and the family members have an interview with both geriatrician and specialist nurse: the physician communicates the diagnosis and shares therapeutic, prognostic, and assistance aspects, and the nurse defines the care strategies, such as non-pharmacological home management of cognitive and behavioral disorders, and gives helpful information on lifestyle and community resources for patients with dementia. During the interview, special emphasis is placed on the importance of attending the course of training and support of family members in order to optimize the management of the disease and improve living with the patient. Finally, to motivate family caregivers to attend the course, it is described as a decisive therapeutic intervention that must be associated with pharmacological therapy. The effectiveness of the family support intervention could be also evident on the low use of medical resources. Recent data from telehealth home support during the COVID-19 confinement for community-dwelling older adults with MCI or mild dementia found that 39% of respondents had contacted health and social services (36), whereas in our sample, only 8.8% of patients have needed a contact.

Another interesting result is that caregiving burden was higher before than after lockdown. This could be due to several reasons: first, it could be a direct effect of the knowledges learned during the course. There are a lot of data in the literature demonstrating that psychoeducational interventions have a strong effect on caregiver's burden: most of the interventions are based on educational programs focused on increasing caregivers' knowledge of the disease and developing specific skills to deal with challenges in caregiving (16), such as in our course. At the first caregiving burden evaluation, performed at the end of the course for family members, the caregivers acquired information that they will have implemented in the months after. Indeed, during the course, we pay great attention to promote caregiver resilience and positive growth, we make participants aware on what community resources are available, we show some simple relaxation techniques that can help relieve stress, we highlight the benefit of physical activity in reducing stress and improving overall well-being, we give information to better understand and cope with the behaviors and personality changes that often accompany AD and other types of dementia, we give contacts for phone and online support, and we give financial and legal information that can be useful for future care and decisions. Second, this result could be related also to the not worsening of neuropsychiatric symptoms that we found and described previously. Research studies have consistently found neuropsychiatric symptoms to be most disturbing to family caregivers (37). In addition, long-term longitudinal studies showed that neuropsychiatric symptoms early during dementia (38), as well as their subsequent worsening (39), were most predictive of increases in burden scores over time. Neuropsychiatric symptoms are distressing for family caregivers because they are unpredictable, disruptive, difficult to manage, potentially embarrassing or abusive, and sleep depriving (37). We did not find a worsening of neuropsychiatric symptoms from the evaluation made before the family course and that after lockdown, and this may have influenced the caregiving burden. Third, the caregiving burden reduction could be the result of lockdown itself. This is the eventuality if the interviewed caregiver was isolated from the patient during the lockdown period and so the caregiver did not have to undergo the behavioral symptoms related to dementia. However, we must note that more than 70% of patients were living with the interviewed caregiver. Moreover, the lockdown may have caused an attention switch to more stressful situations than caregiving itself or the caregiver gets used to the caregiver environment.

Finally, we want to discuss the results on the functional status of our sample of patients: from the first evaluation, made before the family course, and the second one, it has been 8–10 months. During this time, we can suppose a progressive worsening of dementia severity that includes a progressive loss of independence in IADL and BADL, as we found in our data. Interestingly, caregiving burden was associated only to BADL, with higher burden associated with higher loss of BADL, and this data was evident on the results of regression analysis. BADLs consist of self-care tasks that include bathing, grooming, dressing, toilet hygiene, functional mobility, and self-feeding. The impairment of these basic activities increases the personal involvement of the caregiver in terms of the number of daily care hours, leading to higher caregiving burden (40). In a large survey of caregivers in five European countries, BADL impairment emerged as the most problematic area, followed by behavior problems, cognitive impairment, and communication problems (41). Another study showed that functional dependency was more strongly correlated with the number of care hours than neuropsychiatric symptoms and was the only factor independently associated with missing hours at work for those who were employed (42). Based on these data, we can suppose that support actions aimed at lightening the ADL-related workload can also improve caregiving burden.

Before conclusions, some limitations of this study should be acknowledged. First, the sample of the study is not very large; moreover, most variables did not have a normal distribution. Thus, to overcome possible statistical bias, we performed non-parametric statistical analysis. Second, the first evaluation was made by personal interview, whereas the second one was made by phone, and this could be a risk of bias. Third, we cannot collect data on the cognitive status of patients after lockdown due to pandemic restriction. Fourth, the lockdown itself may have determined a low use of medical resources. Finally, a control group of patients whose caregivers did not attend family course is missing.

The strengths of our study include a longitudinal evaluation with the same tests performed close to a family support intervention and after a dramatic event, such as pandemic lockdown.

## Conclusions and Implications

In conclusion, our study demonstrated that a complete family support intervention with the aim to teach, train, and aid caregivers of patients with MCI, AD, and other types of dementia can reduce the caregivers' burden even in a particular negative period, such as the COVID-19 lockdown.

The implementation of non-pharmacological strategies in the treatment of patients with dementia can reduce the use of potentially harmful drugs and improve the quality of life of patients and caregivers.

## Data Availability Statement

The raw data supporting the conclusions of this article will be made available by the authors, without undue reservation.

## Ethics Statement

Ethical review and approval was not required for the study on human participants in accordance with the local legislation and institutional requirements. The patients/participants provided their written informed consent to participate in this study.

## Author Contributions

LC and EM had the initial idea. EM and CC collected the data. LC and FA analyzed the data. LC wrote the first draft. All authors carefully reviewed, discussed and contributed to various draft of the manuscript. All authors approved the final manuscript.

## Conflict of Interest

The authors declare that the research was conducted in the absence of any commercial or financial relationships that could be construed as a potential conflict of interest.
