# Supplementary material for: Effectiveness of a Family Support Intervention on Caregiving Burden in Family of Elderly Patients With Cognitive Decline After the COVID-19 Lockdown
Source: Front Psychiatry. 2021 Mar 4;12:590104. doi: 10.3389/fpsyt.2021.590104 (PMC7971109; doi:10.3389/fpsyt.2021.590104)
Supplement: Supplementary file 1 [file Data_Sheet_1.PDF]

Program of the course

Day one

Introduction of the course and presentation of the Alzheimer’s Center
Cognitive decline, Alzheimer’s disease, and other dementias: clinical presentation, course of the disease, communicating the diagnosis

Speakers: Geriatricians

Day two

Behavioral and Psychological Symptoms of Dementia

Speakers: Geriatrician and Psychologist

Day three

Neuropsychological aspects of Dementia and non-pharmacological treatments

Speaker: Psychologist

Day four

Legal protection of patients with Alzheimer’s disease

Speaker: Lawyer

Day five

How to live with Alzheimer’s disease: strategies for a successful cope with dementia

Speakers: Geriatricians, Psychologists, nurses, rehabilitation specialists

---

Day six

The severe phase of dementia: the role of General Practitioner

Speaker: General Practitioner

Day seven

The family and the Alzheimer's Disease

Speaker: Psychologist

Day eight

Alzheimer's disease help: support groups and community resources

Speakers: social worker, nurse
